# Damping Enhancement of Composite Panels by Inclusion of Shunted Piezoelectric Patches: A Wave-Based Modelling Approach

**DOI:** 10.3390/ma8020815

**Published:** 2015-02-17

**Authors:** Dimitrios Chronopoulos, Manuel Collet, Mohamed Ichchou, Tahir Shah

**Affiliations:** 1 Division of Materials, Mechanics and Structures, University Park, the University of Nottingham, Nottingham NG7 2RD, UK; 2 LTDS, UMR-CNRS 5513, 36 Avenue Guy de Collongue, 69130 Ecully, France; E-Mail: Manuel.collet@ec-lyon.fr; 3 Ecole Centrale de Lyon, 36 Avenue Guy de Collongue, 69130 Ecully, France; E-Mail: mohamed.ichchou@ec-lyon.fr

**Keywords:** damping, wave propagation, piezoelectricity, composite structures

## Abstract

The waves propagating within complex smart structures are hereby computed by employing a wave and finite element method. The structures can be of arbitrary layering and of complex geometric characteristics as long as they exhibit two-dimensional periodicity. The piezoelectric coupling phenomena are considered within the finite element formulation. The mass, stiffness and piezoelectric stiffness matrices of the modelled segment can be extracted using a conventional finite element code. The post-processing of these matrices involves the formulation of an eigenproblem whose solutions provide the phase velocities for each wave propagating within the structure and for any chosen direction of propagation. The model is then modified in order to account for a shunted piezoelectric patch connected to the composite structure. The impact of the energy dissipation induced by the shunted circuit on the total damping loss factor of the composite panel is then computed. The influence of the additional mass and stiffness provided by the attached piezoelectric devices on the wave propagation characteristics of the structure is also investigated.

## 1. Introduction

Complex composite structures are nowadays extensively used within the aerospace, automotive and energy sectors. The analysis of such structures often becomes more complex with the addition of smart material configurations which are used for energy harvesting, vibration control and attenuation as well as for monitoring the structural health of the master structure. Especially with regard to enhancing the vibrational damping properties of the structure, shunted piezoelectric patches can be employed in order to transform and eventually dissipate the strain energy through the connected electrical circuit. Modelling the wave propagation within smart composites would allow for a significant reduction of the computational effort associated with developing and solving numerical finite element models of the entire structure. It would also allow for understanding the impact of the addition of smart layers and patches on the dynamic behavior and the damping properties of the master structure.

The wave propagation within smart structures has been a recently raised subject of research. In [1] the authors used an analytical approach in order to study the propagation of longitudinal waves within a periodic smart rod. The considered smart composite comprised shape memory alloys inserts periodically embedded in the base material. In [2] a spectral finite element formulation was developed for studying the wave propagation characteristics of beams comprising auxetic materials. In [3] the authors used Bloch’s theorem in order to model the wave propagation characteristics of plates having embedded shunted piezoelectric patches. In [4,5] methodologies were developed for optimizing periodic 1D waveguides comprising a distributed network of shunted piezoelectric patches. An experimental investigation of a cantilever beam comprising a shunted piezoelectric periodic array was presented in [6], while experiments on a two dimensional panel with shunted piezoelectric patches were exhibited in [7]. In [8] the propagation of waves in inhomogeneous piezocomposite layered media caused by mechanical loading and electrical excitation was studied using a thin layer approach. Finally in [9] the Wave Finite Element Method (WFEM) was coupled to the FEM in order to compute the wave transmission coefficients of a smart connecting junction.

The analysis of wave propagation within periodic structures was firstly considered in the pioneering work of the author in [10]. The Periodic Structure Theory (PST) was first coupled to the FEM in order to model periodic segments of arbitrary complexity in [11] based on the considerations presented in [12]. The WFEM for two dimensional structures was introduced in [13] in order to further improve the computational efficiency of the approach. It has been successfully applied for computing the dynamic [14–16] as well as the vibroacoustic [17–19] response of layered shell structures and stiffened panels [20,21]. The same approach was used in [22] in order to compute the dynamic response of two dimensional infinite structures. Particular attention has been paid to computing the wave propagation properties [23] as well as the group velocity [24] within structures having frequency-dependent damping and stiffness.

In this paper, a wave and finite element approach is used in order to compute the two dimensional wave propagation characteristics of a periodic structure comprising piezoelectric configurations that are shunted by an **L**–**R** circuit. The structure can be of arbitrary layering and complex geometric characteristics. The impact of the mass and stiffness provided by the attached piezoelectric devices as well as the influence of the piezoelectric coupling itself on the wave propagation characteristics of the structure is subsequently investigated. Last but not least, the frequency-dependent structural damping properties derived from both the piezoelectric shunted patches and the intrinsic damping of the panel are computed.

The paper is organized as follows. In Section 2 the wave and finite element approach for predicting the waves propagating within two-dimensional structures of arbitrary complexity is presented. In Section 3 the equations are reorganized for taking into account the addition of the shunted piezoelectric configurations and the frequency-dependent structural damping properties. In Section 4 a sandwich panel comprising attached piezoelectric patches is considered and numerical results are presented on the wave propagation as well as the structural damping properties. Conclusions on the presented work are eventually given in Section 5.

## 2. Wave Propagation in Complex Periodic Structures

**Figure 1.**
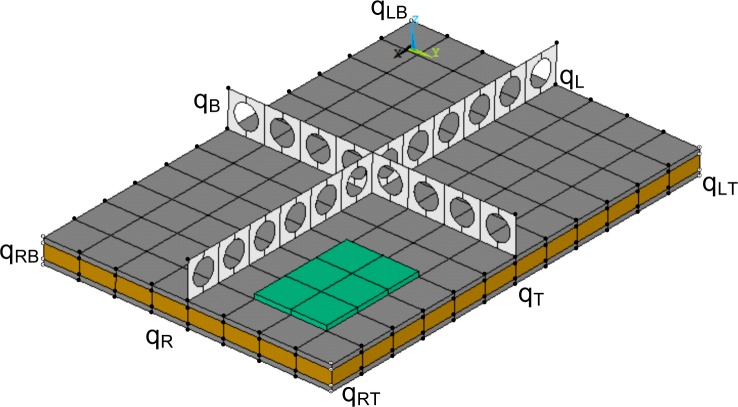
A finite element modelled periodic segment of a generic smart layered stiffened panel.

A two dimensional periodic segment of a layered panel including stiffening and fuzzy attached structures is hereby considered (see Figure 1) with *L_x_*, *L_y_* being its dimensions in the *x* and *y* directions respectively. The segment is modelled using a conventional finite element software. The mass and stiffness matrices of the segment **M** and **K** are extracted and the included degree of freedom (DoF) *q_i_* are reordered according to a predefined sequence such as:

$$q={\left\{q_{I}\;q_{B}\;q_{T}\;q_{L}\;q_{R}\;q_{\mathrm{LB}}\;q_{\mathrm{RB}}\;q_{\mathrm{LT}}\;q_{\mathrm{RT}}\right\}}^{T}$$ (1)

corresponding to the internal, the edge and the vertex DoF (see Figure 1). The free harmonic vibration equation of motion for the modelled segment is written as:

$$[K-\omega^{2}M]q=0$$ (2)

The analysis then follows as in [11] with the ensuing relations being assumed for the displacement DoF under the passage of a time-harmonic wave:

$$\begin{array}{c} q_{R}=e^{-i\varepsilon_{x}}q_{L}=,\;q_{T}=e^{-i\varepsilon_{y}}q_{B}\\ q_{\mathrm{RB}}=e^{-i\varepsilon_{x}}q_{\mathrm{LB}},\;q_{\mathrm{LT}}=e^{-i\varepsilon_{y}}q_{\mathrm{LB}},\;q_{\mathrm{RT}}=e^{-i\varepsilon_{x}-i\varepsilon_{y}}q_{\mathrm{LB}} \end{array}$$ (3)

with ε*_x_* and ε*_y_* being the propagation constants in the *x* and *y* directions, which correspond to a phase lag between the displacement DoF. The wavenumbers *k_x_* and *k_y_* are directly related to the propagation constants through the relation:

$$\varepsilon_{x}=k_{x}L_{x},\;\varepsilon_{y}=k_{y}L_{y}$$ (4)

Considering Equation (3) in tensorial form gives:

$$q=\left[\begin{array}{cccc} I & 0 & 0 & 0\\ 0 & I & 0 & 0\\ 0 & {\mathrm{Ie}}^{-i\varepsilon_{y}} & 0 & 0\\ 0 & 0 & I & 0\\ 0 & 0 & {\mathrm{Ie}}^{-i\varepsilon_{x}} & 0\\ 0 & 0 & 0 & I\\ 0 & 0 & 0 & {\mathrm{Ie}}^{-i\varepsilon_{x}}\\ 0 & 0 & 0 & {\mathrm{Ie}}^{-i\varepsilon_{y}}\\ 0 & 0 & 0 & {\mathrm{Ie}}^{-i\varepsilon_{x}-i\varepsilon_{y}} \end{array}\right]u=\mathrm{Ru}$$ (5)

with u being the reduced set of DoF: u = {q_I_ q_B_ q_L_ q_LB_}*^T^*. The equation of free harmonic vibration of the modelled segment can now be written as:

$$[R^{*}KR-\omega^{2}R^{*}\mathrm{MR}]u=0$$ (6)

with ^*^ denoting the Hermitian transpose. The most practical procedure for extracting the wave propagation characteristics of the segment from Equation (6) is injecting a set of assumed propagation constants ε*_x_*, ε*_y_*. The set of these constants can be chosen in relation to the direction of propagation towards which the wavenumbers are to be sought and according to the desired resolution of the wavenumber curves. When injecting a set of ε*_x_*, ε*_y_* into Equation (6), the equation is transformed into a standard eigenvalue problem and can be solved for the eigenvectors U that describe the deformation of the segment under the passage of each wave type at an angular frequency equal to the square root of the corresponding eigenvalue λ = ω^2^. A complete description of each passing wave including its *x* and *y* directional wavenumbers and its wave shape for a certain frequency is therefore acquired. It is noted that the periodicity condition is defined modulo 2π; therefore solving Equation (6) with a set of ε*_x_*, ε*_y_* varying from 0 to 2π will suffice for a general case scenario. Further considerations on reducing the computational expense of the problem are discussed in [11].

## 3. Accounting for Damping and Piezoelectric Coupling

### 3.1. Inclusion of Shunted Piezoelectric Patches

When a piezoelectric device is included in the segment, considerations related to the piezoelectric coupling and the electric potential of certain nodes have to be done. A finite element discretisation of the strain and electric fields can be directly derived by the piezoelectric constitutive equations [25]:

$$\begin{array}{l} T=c^{E}S_{t}-e^{*}E\\ D=eS_{t}+\epsilon^{S}E \end{array}$$ (7)

where **E** denotes the electric field vector, **T** is the mechanical stress vector; **S**_t_ is the mechanical strain; **D** is the electric displacement vector; **c***^E^* represents the material stiffness matrix under constant electric field; e is the matrix for the direct piezoelectric effect; e^*^ denotes the matrix for the converse piezoelectric effect; and *ϵ^S^* is the permittivity matrix under constant strain.

Three dimensional solid elements with linear interpolation function are hereby considered. The discretised coupled elastoelectric system of equations is generally cast in the form:

$$\begin{array}{l} M_{\mathrm{qq}}\ddot{q}+K_{\mathrm{qq}}q+K_{\mathrm{qv}}v=ℱ\\ K_{\mathrm{qv}}^{*}q+K_{\mathrm{vv}}v=Q \end{array}$$ (8)

with q being the displacement and v the electric potential DoF, **K**_qv_ the part of the coupled system’s stiffness matrix implying piezoelectric coupling, **K**_vv_ the part of the coupled system’s stiffness matrix relating the electric charge to the potential developed within the device, ℱ the external forces applied to the structure and Q the electric charge applied to the nodes of the piezoelectric device. The discretised matrices in Equation (8) can be expressed as

$$\begin{array}{l} M_{\mathrm{qq}}=\underset{V}{∭}N_{q}^{*}\rho N_{q}dV\;K_{\mathrm{qq}}=\underset{V}{∭}B_{q}^{*}c^{E}B_{q}dV\\ K_{\text{qv}}=\underset{V}{∭}B_{q}^{*}e^{*}B_{v}dV\;K_{\mathrm{qq}}=-\underset{V}{∭}B_{v}^{*}\epsilon^{S}B_{v}dV \end{array}$$ (9)

with **N**_q_ and **N**_v_ being the shape functions for the elastic and the electric field respectively, $B_{v}=\nabla N_{v}D$ and $B_{q}={D\text{N}}_{q}$ with D being a linear differential operator typically used in finite element formulation.

Following the electrode definitions mentioned in [26], the electrical potential DoF in the piezoelectric patches are partitioned into three groups:

- For nodes on the outer surfaces of the piezoelectric patches, their associated electrical DoF are called **v**_p_ and they all have the same electrical potential.
- For nodes on the inner surfaces of the piezoelectric patches bonded to the composite structure, their associated electrical DoF are called **v**_g_, and they are grounded.
- For nodes inside the piezoelectric patches, their associated electrical DoF are called **v**_i_.

Equation (8) can therefore be recast in the form

$$\left[\begin{array}{cccc} M_{\mathrm{qq}} & 0 & 0 & 0\\ 0 & 0 & 0 & 0\\ 0 & 0 & 0 & 0\\ 0 & 0 & 0 & 0 \end{array}\right]\left\{\begin{array}{c} \ddot{\text{q}}\\ {\ddot{v}}_{i}\\ {\ddot{v}}_{p}\\ {\ddot{v}}_{g} \end{array}\right\}+\left[\begin{array}{cccc} K_{\mathrm{qq}} & K_{\mathrm{qi}} & K_{\mathrm{qp}} & K_{\text{qg}}\\ K_{\mathrm{qi}}^{*} & K_{\mathrm{ii}} & K_{\mathrm{ip}} & K_{\mathrm{ig}}\\ K_{\mathrm{qp}}^{*} & K_{\mathrm{ip}}^{*} & K_{\text{pp}} & K_{\text{pg}}\\ K_{\text{qg}}^{*} & K_{\mathrm{ig}}^{*} & K_{\text{pg}}^{*} & K_{\text{gg}}^{*} \end{array}\right]\left\{\begin{array}{c} q\\ v_{i}\\ v_{p}\\ v_{g} \end{array}\right\}=\left\{\begin{array}{c} ℱ\\ Q_{i}\\ Q_{p}\\ Q_{g} \end{array}\right\}$$ (10)

As aforementioned, **v**_g_ = 0. Thus, the fourth equation and fourth column in the mass and stiffness matrices can be eliminated. Assuming that there no internal electric charges, therefore $Q_{i}=0$. The internal potential DoF can thus be determined by an exact condensation from Equation (10)

$$v_{i}=-K_{\mathrm{ii}}^{-1}K_{\mathrm{qi}}^{*}q-K_{\mathrm{ii}}^{-1}K_{\mathrm{ip}}v_{p}$$ (11)

As all the nodes on the potential electrode surfaces have identical potentials, a transformation matrix **T**_m_ can be used to define the master potential DoF **v**_m_ as

$$v_{p}=T_{m}v_{m}$$ (12)

and therefore Equation (10) becomes

$$\left[\begin{array}{cc} M_{\mathrm{qq}} & 0\\ 0 & 0 \end{array}\right]\left\{\begin{array}{c} \ddot{\text{q}}\\ {\ddot{\text{v}}}_{m} \end{array}\right\}+\left[\begin{array}{cc} H_{\mathrm{qq}} & H_{\mathrm{qm}}\\ H_{\mathrm{qm}}^{*} & H_{\mathrm{mm}} \end{array}\right]\left\{\begin{array}{c} q\\ v_{m} \end{array}\right\}=\left\{\begin{array}{c} ℱ\\ Q_{m} \end{array}\right\}$$ (13)

with

$$\begin{array}{l} H_{\mathrm{qq}}=K_{\mathrm{qq}}-K_{\mathrm{qi}}K_{\mathrm{ii}}^{-1}K_{\mathrm{qi}}^{*}\\ H_{\mathrm{qm}}=(K_{\mathrm{qq}}-K_{\mathrm{qi}}K_{\mathrm{ii}}^{-1}K_{\mathrm{ip}})T_{m}\\ H_{\mathrm{mm}}=T_{m}^{*}(K_{\mathrm{qq}}-K_{\mathrm{ip}}^{*}K_{\mathrm{ii}}^{-1}K_{\mathrm{ip}})T_{m}\\ Q_{m}=T_{m}^{*}Q_{p} \end{array}$$ (14)

The shunt **R**–**L** circuit is then considered with the electric impedance *Z_s_* of the circuit under harmonic excitation written as

$$Z_{s}=R+i\omega L$$ (15)

The quantities *Q_m_* and *v_m_* can now be expressed as scalar due to the fact that a master DoF is taken into account for the electric potential of the outer nodes. The electric current flowing within the shunt circuit *I_s_* is therefore expressed as

$$I_{s}=i\omega Q_{m}=\frac{v_{m}}{Z_{s}}$$ (16)

By substituting Equation (16) into Equation (13), the electrical DoF can be condensed and the equation that governs the structural dynamics under harmonic excitation is derived

$$[H_{\mathrm{qq}}-\omega^{2}M_{\mathrm{qq}}+H_{\mathrm{qm}}{\left(\frac{1}{i\omega Z_{s}}-H_{\mathrm{mm}}\right)}^{-1}H_{\mathrm{qm}}^{*}]q={ℛ}^{s}q=ℱ$$ (17)

which gives a full finite element based description of the composite structure along with the shunted piezoelectric inclusions through the dynamic stiffness matrix of the ensemble *R^s^*. The configuration is considered to be shunted through an **R**–**L** circuit, which is in series with the capacitance of the piezoelectric patch. The inductance of the circuit is therefore chosen in order to tune the shunted circuit at its resonant frequency, which is

$$f_{t}=\frac{1}{2\pi \sqrt{LC_{pz}^{S}}}$$ (18)

with *L* being the inductance of the circuit; and $C_{pz}^{S}$ is the capacitance of the piezoelectric patch in the thickness direction measured at constant strain and given by

$$C_{pz}^{S}=\frac{\epsilon_{z}^{S}A_{z}}{h_{p}}$$ (19)

with *A_z_* being the area of the surface of the piezoelectric patch perpendicular to *z*-axis; and *h_p_* is the thickness of the patch.

### 3.2. Structural Damping Loss Factor

Taking a look at Equation (17), it is clear that the eigenvalue problem to be solved is of different nature compared with the one exhibited in Equation (6). Assuming free vibration and a general case of a viscous damping matrix **C**_qq_ for the composite structure implies that the eigenvalue problem is modified according to the dynamic stiffness matrix of the ensemble ℛ*^s^*

$$[H_{\mathrm{qq}}-\omega^{2}M_{\mathrm{qq}}+i\omega C_{\mathrm{qq}}{+H}_{\mathrm{qm}}{\left(\frac{1}{i\omega Z_{s}}-H_{\mathrm{mm}}\right)}^{-1}H_{\mathrm{qm}}^{*}]q={ℛ}^{s}q=0$$ (20)

which leads to the modified eigenvalue problem

$$[R^{*}KR-\omega^{2}R^{*}MR]q=0$$ (21)

with

$$\begin{array}{c} M=M_{\mathrm{qq}}\\ K=H_{\mathrm{qq}}+i\omega C_{\mathrm{qq}}+H_{\mathrm{qm}}{\left(\frac{1}{i\omega Z_{s}}-H_{\mathrm{mm}}\right)}^{-1}H_{\mathrm{qm}}^{*} \end{array}$$ (22)

It is now evident that the new matrix K is frequency-dependent, which impedes the direct implementation of a linear eigenvalue solution algorithm. An iterative approach will therefore be applied in order to solve for the resulting eigenvalues and the eigenvectors of Equation (21). An initial estimation for the complex pulsation ω*_j_* is injected in Equation (21). Once again the set of propagation constants ε*_x_*, ε*_y_* are chosen in relation to the direction towards which the wavenumbers are to be sought. The new estimations of ω are given directly by the computed eigenvalues λ*_j_* as $\omega_{j+i}=\sqrt{\lambda_{j}}$ until successive solutions for a certain eigenvalue converge to a desired level.

The computed eigenvalues λ = ω^2^ will also have an imaginary part as ω = ω*_r_* + iω*_i_* with | ω*_i_* |> 0 implying complex values for the wavenumbers of the propagating wave types, otherwise interpreted as spatially decaying motion (for a physical explanation see [24,27,28]) and from which the loss factor of each computed wave type *n* can directly be determined [29] as

$$\eta_{n}(\omega ,\theta )=2\frac{\omega_{i}\omega_{r}}{\omega_{r}^{2}-\omega_{i}^{2}}$$ (23)

with η*_n_*(ω*,* θ) being the loss factor for the wave type *n* at a certain angular frequency ω and propagating towards a certain direction θ. The total frequency-dependent loss factor of a certain wave type can be computed as

$$\eta_{n}(\omega )=\frac{\int_{0}^{2\pi }\eta_{n}(\omega ,\theta )d\theta }{\int_{0}^{2\pi }d\theta }$$ (24)

which can be evaluated at the entire spectrum of interest.

## 4. Numerical Examples

**Figure 2.**
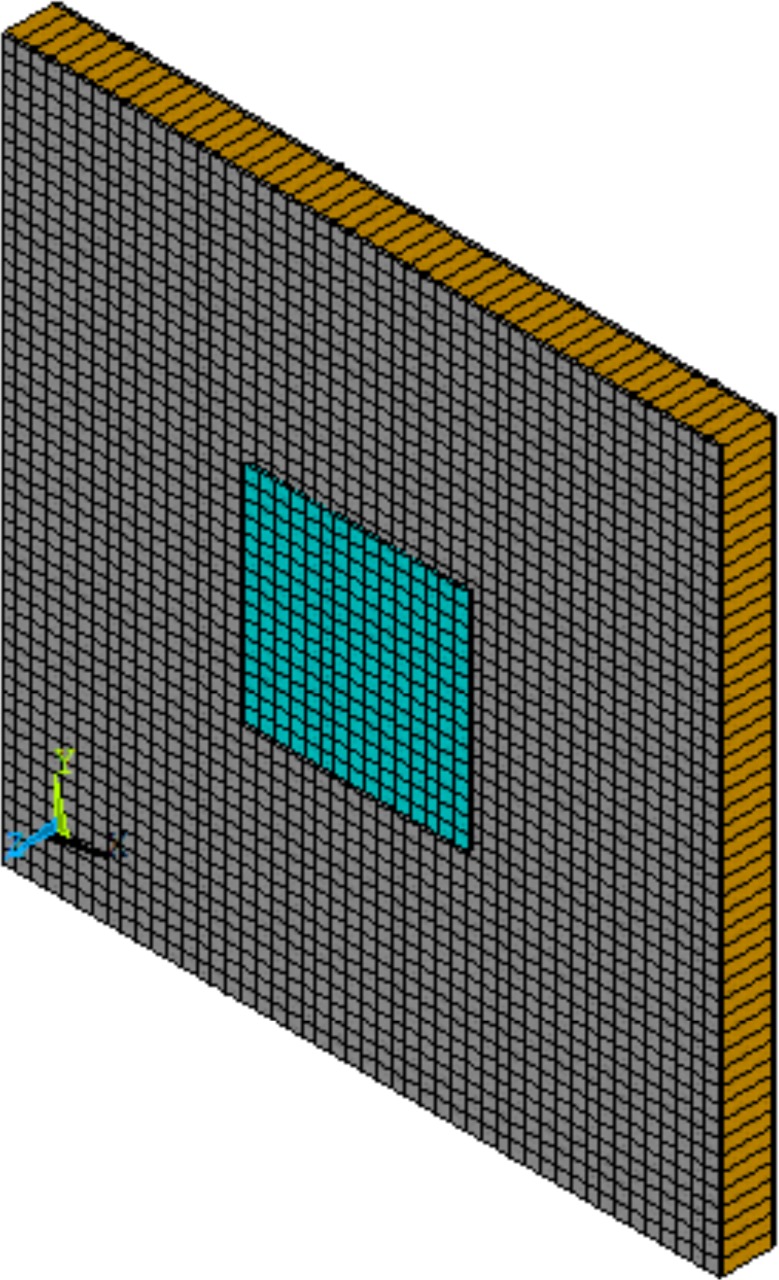
A discretised periodic segment of the modelled sandwich panel with a piezoelectric patch attached to it.

The periodic structure to be modelled is a sandwich panel (see Figure 2) having facesheets made of Material I and a core made of Material II (see Table 1). The thicknesses of the facesheets and the core are equal to *h_f_* = 0.5 mm and *h_c_* = 6.35 mm respectively. The periodicity of the panel in both the *x* and *y* directions is equal to 96 mm. The periodic segment of the panel has a piezoelectric device attached to its upper facesheet. The device is made of Material III and its dimensions are equal to 32 mm × 32 mm × 0.5 mm in length, width and thickness respectively. The piezoelectric stress coupling matrix for the device’s material is equal to:

$$e=\left[\begin{array}{cccccc} 0 & 0 & 0 & 0 & 25.76 & 0\\ 0 & 0 & 0 & 25.76 & 0 & 0\\ -12.374 & -12.374 & 25.6 & 0 & 0 & 0 \end{array}\right]N/(V.m)$$ (25)

while the absolute permittivity matrix under constant strain *ε^S^* is:

$$\varepsilon^{S}=10^{-8}\times \left[\begin{array}{ccc} 2.022 & 0 & 0\\ 0 & 2.022 & 0\\ 0 & 0 & 1.182 \end{array}\right]C/(V.m)$$ (26)

### 4.1. Results on Wave Propagation

**Figure 3.**
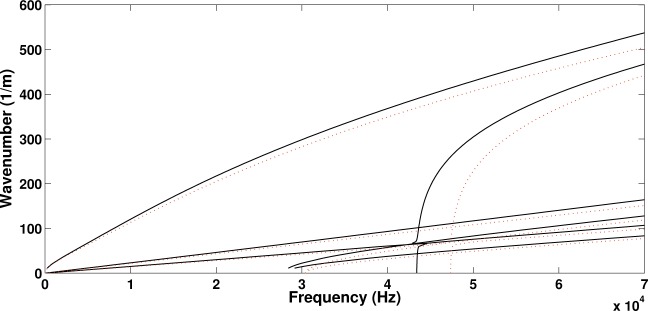
The real part of the wavenumbers propagating at a direction of 45° within the sandwich panel: sandwich panel with no attached patch (⋯), structure with the patch attached to the upper facesheet (piezoelectric coefficients set to zero) (−).

**Figure 4.**
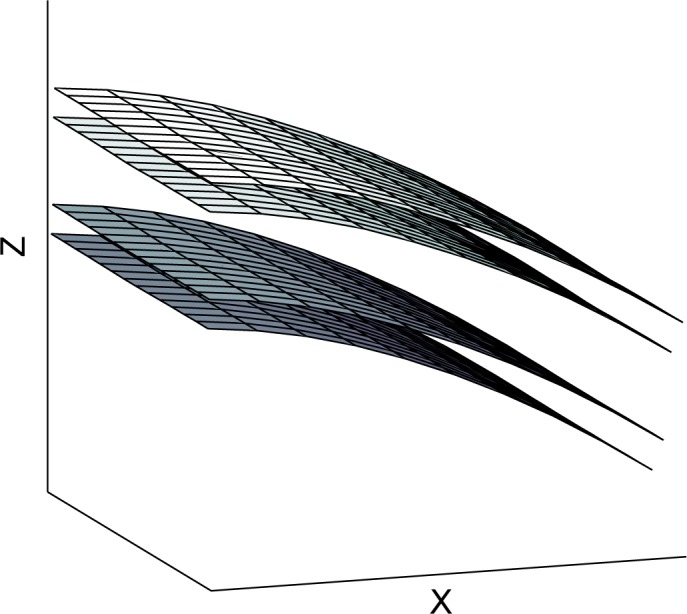
The computed real part of the propagating first flexural wave mode shape. The corresponding depicted wavenumber in the *x* direction is *k_x_* = 7.85 rad/m.

Initially the panel was modelled and solved without the piezoelectric device attached to it. In order to investigate the effect of the added mass and stiffness of the patch on the wave propagation properties of the structure, a second configuration is hereby considered by setting all piezoelectric coupling constants of the attached patch to zero (thus making it behave as a purely structural element). The computed real wavenumbers propagating within the periodic structure towards the direction of 45° are shown in Figure 3 for the two configurations. In Figure 4 the relative displacement of the four surfaces (upper, lower as well as two internal core surfaces) of the sandwich structure under the passage of the first flexural wave mode shape is depicted.

It is observed that in both cases six wave types propagate within the panel up to 70 kHz. When modelling only the layered panel, veering of the dispersion curves is observed at about 47.5 kHz where the curves would otherwise cross (see [30] for more discussion on veering effects). This critical frequency is significantly shifted when the piezoelectric device is added by approximately 5 kHz. It can generally be observed that the wavenumber curves are shifted upwards when the piezoelectric device is added to the panel. This implies that the added mass provided by the device prevails over the added stiffness, shifting the resonances of the panel to lower frequencies. The maximum divergence between the two wavenumber sets is equal to 8.4% at 70 kHz for the flexural wave type.

The third configuration that was modelled and solved included the attached piezoelectric patch having full piezoelectric coupling properties. The results are compared with the ones computed above for when the piezoelectric coefficients of the patch are set to zero in order to observe the effect of the piezoelectric coupling phenomenon itself to the waves propagating within the structure.

**Figure 5.**
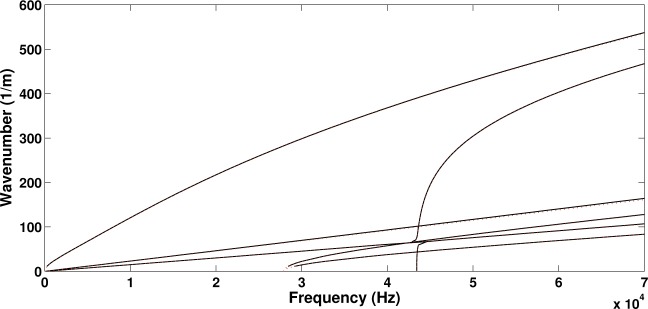
The real part of the wavenumbers propagating at a direction of 45° within the sandwich panel having a piezoelectric patch attached to it: patch with no piezoelectric coupling (−), piezoelectric patch (- -).

It is observed in Figure 5 that the piezoelectric coupling tends to have an insignificant effect on the wavenumbers propagating within the structure as well as to the critical veering frequency. The maximum divergence between the two wavenumber sets is equal to 0.75% at 70 kHz for the flexural wave type. It can therefore be concluded that the effect induced by the added structural mass of the device prevails over the added stiffness provided by the piezoelectric coupling phenomenon.

### 4.2. Results on the Loss Factor of the Panel

**Figure 6.**
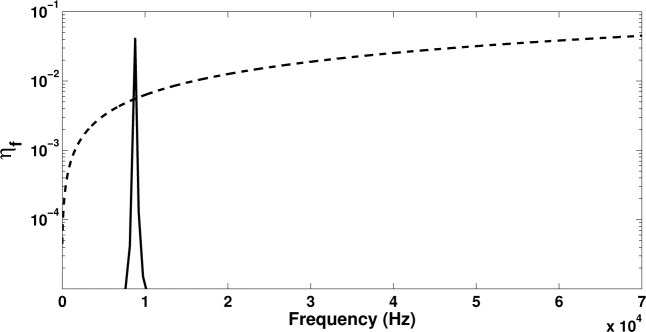
The frequency-dependent loss factor for the flexural wave propagating within the laminate with: a shunted piezoelectric patch (−) and with a viscous damping of C_qq_ = 10^−7^ Kqq (- -).

In this section, the piezoelectric device attached to the laminate will be considered to be shunted through an **R**–**L** circuit tuned to have a resonance frequency at approximately 8 kHz. In order to compare the results to the damping induced by the intrinsic characteristics of the laminate, a viscous type damping matrix with **C**_qq_ = 10^−7^ **K**_qq_ is also considered hereby. In Figure 6 the eigenproblem presented in Equation (21) is solved initially with **H**_qm_ = 0 leaving the intrinsic damping as the only dissipation mechanism. Subsequently, **C**_qq_ is set equal to zero in order to account only for the energy dissipated within the **R**–**L** circuit. Results on the loss factor for the flexural wave η*_f_* are exhibited.

**Figure 7.**
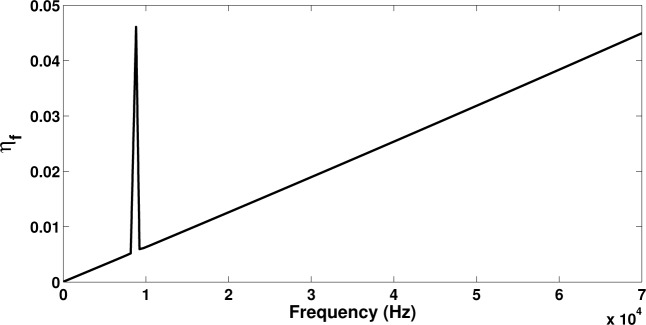
The frequency-dependent total loss factor for the flexural wave propagating within the laminate with a shunted piezoelectric patch and **C**_qq_ = 10^−7^ **K**_qq_.

It is observed that the damping provided by the shunted piezoelectric patch presents an intense peak around the tuning frequency of the circuit, which is approximately an order of magnitude greater than the value of the loss factor for the viscous damping case. On the other hand the intrinsic viscous damping provides more broadband energy dissipation increasing with frequency. In Figure 7 it can be seen that when both sources of dissipation are applied, the dominant damping mechanism around the **R**–**L** tuning frequency will again be the piezoelectric shunt. It can therefore be concluded that when energy dissipation in a broadband frequency range is needed, the intrinsic structural damping solution would be more beneficial; however, when high energy dissipation is needed at targeted frequency ranges, the design of shunted piezoelectric devices can be successfully employed. Broadening the spectrum of the loss factor peak when shunted piezoelectric devices are used is a subject of further investigation. Implementing shunted **R**–**L** circuits in parallel to each other, for example, can result in multi-resonant circuits and can thus efficiently extend the frequency range within which the piezoelectric device will contribute to the total loss factor of the laminate.

## 5. Conclusions

A wave-based approach for predicting the wave propagation characteristics of two dimensional periodic panels comprising piezoelectric devices was hereby presented. The shunting of the piezoelectric patched was also accounted for and the expressions for the loss factor of the panel were derived. Summarising the most important findings of the work:

1. It was observed that the inclusion of a piezoelectric device can have a significant impact on the wavenumbers and therefore the phase and group velocities of the waves propagating within the structure. The critical frequencies at which the wavenumber curves veer apart were also found to be much affected by the addition of the piezoelectric configuration. This impact is directly related to the additional mass and stiffness provided by the piezoelectric device to the structure. In both elaborated examples it was shown that the added mass overcame the added stiffness to shift the wavenumber curves upwards and the resonances of the panel to lower frequencies. This however is not a general conclusion and the impact can vary for different wave types and different structures.
2. The influence of the added stiffness provided by the piezoelectric coupling phenomenon on the wavenumbers was investigated. It is found that for conventional piezoelectric materials this influence is negligible compared with the effects induced by the added structure of the device.
3. Shunting the piezoelectric device through an R–L circuit was exhibited to provide a significant increase of the structural loss factor of the panel around the tuning frequency of the circuit. It was shown that while increasing the intrinsic structural damping is beneficial in a broadband frequency range, when high damping is needed at targeted frequency ranges, the solution of shunted piezoelectric devices can be successfully employed. Further work will be focused on broadening the spectrum of the damping increase by implementing multi-resonant parallel circuits.

## Figures and Tables

**Table 1.** Mechanical properties of materials.

Material I	Material II	Material III
ρ = 1600 kg/m^3^	ρ = 160 kg/m^3^	ρ = 7650 kg/m^3^
*v* = 0.15	*v* = 0.18	*v* = 0.10
*E* = 49 GPa	*E* = 0.258 GPa	*E* = 119 GPa
